# Characterization of Lactate Metabolism Score in Breast and Thyroid Cancers to Assist Immunotherapy via Large-Scale Transcriptomic Data Analysis

**DOI:** 10.3389/fphar.2022.928419

**Published:** 2022-07-07

**Authors:** Cheng Wang, Zheng Qu, Li Chen, Yunhao Pan, Yiqing Tang, Guangfu Hu, Ran Gao, Ruijie Niu, Qiang Liu, Xingyan Gao, Yi Fang

**Affiliations:** ^1^ Department of Breast Surgery, Huangpu Branch, Shanghai Ninth People’s Hospital, Affiliated to Shanghai Jiao Tong University School of Medicine, Shanghai, China; ^2^ Department of Breast Surgical Oncology, National Cancer Center/National Clinical Research Center for Cancer/Cancer Hospital, Chinese Academy of Medical Sciences and Peking Union Medical College, Beijing, China; ^3^ Department of Thyroid and Breast Surgery, Tongji Hospital, Tongji Medical College of Huazhong University of Science and Technology, Wuhan, China

**Keywords:** breast cancer, thyroid cancer, cancer immunotherapy, immune infiltrates, lactate metabolism score, prognostic factor

## Abstract

Breast cancer (BC) and thyroid cancer (TC) have the highest rate of incidence, especially in women. Previous studies have revealed that lactate provides energetic and anabolic support to cancer cells, thus serving as an important oncometabolite with both extracellular and intracellular signaling functions. However, the correlation of lactate metabolism scores with thyroid and breast cancer immune characteristics remains to be systematically analyzed. To investigate the role of lactate at the transcriptome level and its correlation with the clinical outcome of BC and TC, transcriptome data of 1,217 patients with breast cancer (BC) and 568 patients with thyroid cancer (TC) were collected from The *Cancer* Genome Atlas (TCGA) and Gene Expression Omnibus (GEO) datasets with their corresponding clinical and somatic mutation data. The lactate metabolism score was calculated based on a single-sample gene set enrichment analysis (ssGSEA). The results showed that lactate metabolism-related genes and lactate metabolism scores was significantly associated with the survival of patients with BRCA and THCA. Notably, the lactate metabolism scores were strongly correlated with human leukocyte antigen (HLA) expression, tumor-infiltrating lymphocyte (TIL) infiltration, and interferon (IFN) response in BC and TC. Furthermore, the lactate metabolism score was an independent prognostic factor and could serve as a reliable predictor of overall survival, clinical characteristics, and immune cell infiltration, with the potential to be applied in immunotherapy or precise chemotherapy of BC and TC.

## 1 Introduction

Breast cancer (BC) and thyroid cancer (TC) are two malignant diseases with a high rate of occurrence in women (Siegel et al., 2022). In 2020, BC surpassed lung cancer as the most commonly diagnosed cancer, with an estimated 2.3 million new cases. TC affects 586,000 people globally, and the global incidence rate in females is three times greater than in males (Sung et al., 2021). Due to tumor heterogeneity, treatment resistance, metastasis, and disease recurrence, comprehensive treatment of BC and TC is associated with numerous challenges (Waks and Winer, 2019; Nabhan et al., 2021). Both thyroid and breast tissues are related to hormones, and changes in the endocrine system are closely related to the development of thyroid and breast tumors. Moreover, a family history of breast cancer, estrogen receptor (ER) status, progesterone receptor (PR) status, and triiodothyronine levels are associated with the development of thyroid and breast tumors (Kuo et al., 2016; Li et al., 2019). Simultaneously, BC with TC is more common, and the incidence is gradually on the rise worldwide. Studies have shown that when the first primary cancer is TC, the most common second primary cancer is BC; similarly, TC is also the most commonly diagnosed secondary malignant tumor during BC recurrence (Vassilopoulou-Sellin et al., 1999). The occurrence of BC with TC is common among patients with multiple primary cancers (Endo et al., 2018). Therefore, it is imperative to screen new potential indices, especially immunotherapy-associated parameters, for prognostic prediction and individualized treatment of patients with BC and TC.

Lactate, which has been long neglected as a waste product derived from glycolysis, is now regarded as an important carbon source for cellular metabolism. It plays a key role in tumor development, metastasis, immunosuppression, and therapeutic response (García-Cañaveras et al., 2019; Ippolito et al., 2019). Studies have demonstrated that the glycolytic and glutaminolytic pathways are major contributors to lactate accumulation in the tumor microenvironment (TME) (Yang et al., 2017; Lee et al., 2018; Nagao et al., 2019). Several oncogenes and tumor suppressor genes, including *Myc*, epidermal growth factor (*EGF*), phosphoinositol three kinase (*PI3K*), mTOR, and hypoxia-inducible factor 1α (*HIF-1α*), are involved in the metabolic switch from oxidative phosphorylation (OXPHOS) toward altered glycolysis of tumor cells (Dang, 2012; Lu et al., 2015). The target of HIF-1α is monocarboxylate transporter (MCT), which ensures both adequate glucose delivery into the cell and secretion of accumulated lactate out of the cell, and excessive levels of lactate produced by cancer cells are removed by MCTs (de la Cruz-López et al., 2019). Accordingly, lactate transport by MCTs represents a therapeutic vulnerability for cancer cells (Payen et al., 2020). Moreover, lactate and glutamine support NADPH production via isocitrate dehydrogenase 1 (IDH1) and malic enzyme 1 (ME1), respectively, under glucose-deprived conditions, and ME1 can synergize with mitochondrial IDH2 to maintain antioxidant systems to support tumor growth and metastasis (Shao et al., 2020; Ying et al., 2021). In summary, the accumulation of lactate in solid tumors is a pivotal and early event in the development of malignancies, and targeting lactate metabolism is considered a promising cancer therapeutic strategy.

Immunotherapy has improved the prognosis of a variety of cancers, and it forms the cornerstone of cancer treatment alongside traditional surgery, chemotherapy, radiotherapy, and targeted therapy (Kruger et al., 2019). However, the immunosuppressive roles of lactate accumulation within the TME limit the efficacy of immunotherapy. Several experiments have shown that lactate is an effective antitumor T-cell inhibitor that facilitates the development of tumor-permissive (i.e., immunosuppressive) T regulatory cells (Tregs). Kelderman et al. concluded that in patients with advanced cutaneous melanoma, the long-term benefit of ipilimumab treatment was unlikely for patients with baseline serum LDH levels greater than twice the upper limit of normal levels (Kelderman et al., 2014). Zhang et al. found that serum LDH levels predicted response to immune checkpoint inhibitor therapy—progression-free and significantly shorter overall survival (OS) in patients with high pretreatment LDH levels in non-small cell lung cancer (Zhang et al., 2019). Additionally, research showed that raising intratumoral pH improved CTL infiltration and response to immunotherapy against CTLA-4, anti-PD-1, and adoptive cell transfer in mouse melanoma and pancreatic cancer models (Pilon-Thomas et al., 2016). However, it the role of lactate in the response to immune checkpoint inhibition in BC and TC is not understood. Thus, it is challenging but necessary to identify a better predictor to evaluate the clinical outcomes accurately before prescribing immunotherapy for patients with BC and TC.

To our knowledge, this is the first study to develop a lactate metabolism score system and investigate its role in both BC and TC. Moreover, we analyzed the relationship between the lactate metabolism score and the immune microenvironment features and response to immunotherapy. We also assessed the difference in susceptibility to common antineoplastic agents between the high and low lactate metabolism score groups using the GDSC database. These findings might aid with immune-targeted therapy by providing an alternative signature to predict prognosis and treatment success in BC and TC.

## 2 Materials and Methods

### 2.1 Data Acquisition and Preliminary Analysis

The expression profile data TCGA-BRCA and TCGA-THCA for breast invasive and thyroid cancers, respectively, were downloaded from the UCSC Xena database (http://xena.ucsc.edu/) with the data type “count” and the count value normalized to the TPM value. TCGA-BRCA contained transcriptomic data from a total of 1,217 patients with BC, including 1,072 primary tumor samples (01A) and 99 normal samples (11A); TCGA-THCA contained transcriptomic data from a total of 568TC cases, including 497 (01A) primary tumor samples and 56 (11A) normal samples, which were included in this analysis.

“Masked Somatic Mutation” data were selected from the TCGA GDC official website (https://portal.gdc.cancer.gov/) as somatic mutation data for patients with invasive BC (n = 1,044) and TC (n = 496), and the data were pre-processed using VarScan software. Data on somatic mutations were visualized using the “maftools” R package (Mayakonda and Koeffler, 2016). Meanwhile, to analyze gene copy number changes in patients with TCGA-BRCA and TCGA-THCA, the patient’s “Masked Copy Number Segment” data were downloaded using the “TCGAbiolinks” R package (Colaprico et al., 2016).

In addition, the patient clinical data (phenotype), including age, survival state, follow-up time, and cancer stage, matching TCGA-BRAC and TCGA-THCA were accessed and downloaded from the UCSC Xena database. Finally, combined with the mutation information and clinical information matching of patients, the clinical data of 1,072 patients with BRCA and 497 with THCA were included.

The microarray data set consisted of four BC datasets, GSE36295 (Merdad et al., 2014), GSE109169 (Chang et al., 2018), GSE58812 (Jézéquel et al., 2015), and GSE20685 (Kao et al., 2011); and 1 TC dataset, GSE165724 (He et al., 2021). GSE36295, GSE106916, and GSE20685 were used to verify the lactate metabolism score, and GSE58812 and GSE20685 were used to verify the prognosis of BC. Microarray data details are presented in Table 1.

**TABLE 1:** Common datasets used in this study.

Tumor Types	Datasets	Samples	
		Tumor	Normal
BRCA	GSE36295	45	5
	GSE109169	25	25
	GSE58812	107	0
	GSE20685	327	0
	TCGA-BRCA	1,072	99
THCA	GSE165724	58	16
	TCGA-THCA	497	56

### 2.2 Collection and Categorization of Characteristic Gene Set

245 Lactate metabolism-related genes were obtained from MSigDB database (Liberzon et al., 2011) “GOMF_LACTATE_DEHYDROGENASE_ACTIVITY”, “GOBP_LACTATE_TRANSMEMBRANE_TRANSPORT”, “HP_INCREASED_SERUM_LACTATE”, “HP_INCREASED_LACTATE_DEHYDROGENASE_LEVEL”, “HP_ABNORMAL_LACTATE_DEHYDROGENASE_LEVEL”, “GOMF_LACTATE_TRANSMEMBRANE_TRANSPORTER_ACTIVITY”, and “GOMF_L_LACTATE_DEHYDROGENASE_ACTIVITY”.

The gene sets of HLA, TILs, immune cytolytic activity (CYT), and interferon (IFN) were obtained from previous studies (Ju et al., 2021). All of the above gene set details are presented in Supplementary Table S1.

### 2.3 Evaluation of Lactate Metabolism Score

To quantify lactate metabolism in BC and TC, the lactate metabolism score was calculated based on a single-sample gene set enrichment analysis (ssGSEA) (Hänzelmann et al., 2013), using the gene set related to the lactate metabolism score (Supplementary Table S1). The lactate metabolism scores between tumor and normal samples were estimated from the TCGA and GEO datasets. According to the median value of the lactate metabolism score, the patients with a score above the median value were classified as the high lactate metabolic group, and those with a score below the median value were classified as the low lactate metabolic group.

### 2.4 Survival Analysis

We compared the OS and progression-free survival (PFS) of patients with cancer between the high and low lactate groups in BC and TC. Kaplan-Meier (K-M) analyses were performed to compare the differences in survival time (Bland and Altman, 1998). The log-rank test was applied, and the *p*-value was calculated. Statistical significance was set at *p* < 0.05.

To evaluate the predictive ability of lactate-related gene expression for the prognosis of different tumors, single factor Cox regression and Lasso regression analyses were performed. Then, multivariate Cox regression analysis was applied to further obtain the prognostic characteristic genes related to lactate metabolism based on TCGA-BRCA and TCGA-THCA datasets. First, the relationship between the expression of each differential expressed gene and OS and PFS was analyzed using univariate Cox proportional regression analysis, and genes with *p* < 0.05 were retained. Next, the Lasso algorithm was used to screen for some meaningful variables in the univariate Cox regression analysis. To obtain more accurate independent prognostic factors (prognostic characteristic genes), a final screening was performed using multivariate Cox regression analysis.

### 2.5 Assessment of Immune Infiltration

To evaluate the association of the lactate metabolism score with thyroid and breast cancer, the tumor’s HLA, CYT, IFN, and TIL scores were calculated by ssGSEA. Then, the relationship between the scores and the lactate metabolism scores was analyzed using Spearman’s correlation. In addition, the macrophage and CD4 cell scores were calculated according to the ssGSEA algorithm (Hänzelmann et al., 2013). Finally, the macrophage and CD4 cell infiltration were compared between high and low lactate metabolism scorescore groups.

### 2.6 Evaluation of Tumor Immune Activity, Tumor Mutation Burden, and Immunologic Characteristics

To assess the tumor immune activity, stromal and immune cells in malignant tumors were estimated using expression data based on the ESTIMATE algorithm (Yoshihara et al., 2013). The algorithm generated an immune score for each tumor sample and quantified the immune activity (immune infiltration level) of the tumor based on the samples of immune gene expression. For each tumor sample, the tumor mutation burden (TMB) was determined as the total number of somatic mutations (other than silent mutations) detected in the tumor (Merino et al., 2020). To compare the ability of immune prediction of lactate metabolism scores, Each tumor sample was studied based on a set of glycolytic genes of ssGSEAlactate metabolism score.

### 2.7 Gene Set Enrichment Analysis

To explore the potential biological functions of the gene sets related to lactate metabolism between the low and high groups in BC and TC, GSEA was performed based on a carefully selected gene set “c2. cp.kegg.v7.2. symbols” (Hänzelmann et al., 2013). Values with *p* < 0.05 were considered statistically significant.

### 2.8 Drug Sensitivity Analysis

The Genomics of Drug Sensitivity in *Cancer* (GDSC) (www.cancerrxgene.org/) database can be used to obtain tumor drug response data and sensitive genomic biomarkers (Yang et al., 2013). Based on the gene expression profile, a ridge regression model was constructed using the pRRophetic algorithm (Geeleher et al., 2014). The susceptibility to common anticancer drugs in the high and low lactate groups was predicted based on the IC50 values.

The potential therapeutic response of ICB to tumor therapy was projected through the tumor immune dysfunction and exclusion (TIDE) score, which is a computational algorithm based on gene expression profiles (http://tide.dfci.harvard.edu) (Fu et al., 2020). According to the results of the TIDE analysis, the differences in various indicators of tumor immunotherapy, such as TIDE, CD8, and CD274, were compared between the high and low lactate groups.

### 2.9 Statistical Analysis

Data were analyzed in R software (Version 4.0.2). To compare continuous variables between two groups, the statistical significance of normally distributed variables was estimated by a Student’s *t*-test. Differences in non-normally distributed variables across groups were analyzed by the Mann-Whitney U test (i.e., the Wilcoxon rank-sum test). The chi-square test or Fisher’s exact test was used to compare and analyze the statistical significance between the two groups of categorical variables.

The correlation between the two variables was calculated using the Spearman method. Statistical significance was set at *p* < 0.05 (Spearman correlation test). The R package “survivalROC” was used to plot the receiver operating characteristic (ROC) curve (Blanche et al., 2013), and the area under the ROC curve (AUC) was applied to assess the prognostic performance of the lactate metabolism score.

## 3 Results

### 3.1 Comparison of Lactate Metabolism Scores and Expression of Lactate Metabolism-Related Genes in Breast Cancer and Thyroid Cancer

**FIGURE 1:**
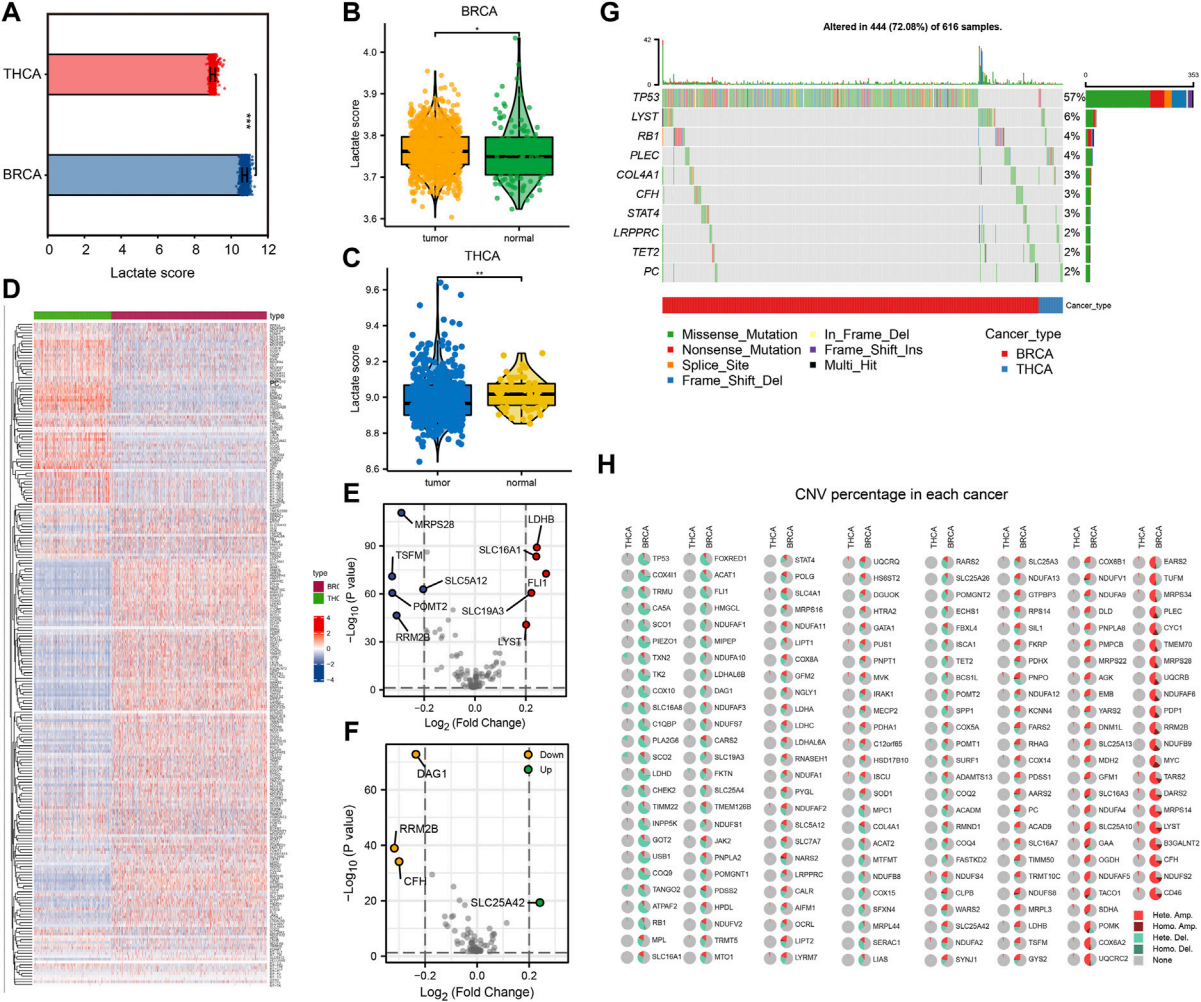
Comparison of lactate metabolism score and expression of lactate metabolism-related genes in breast cancer (BC) and thyroid cancer (TC). **(A)** Comparison of lactate metabolism scores in BC and TC. **(B)** Comparison of lactate metabolism scores between normal and neoplastic tissues in BC. **(C)** Comparison of lactate metabolism scores between normal and neoplastic tissues in TC. **(D)** The expression of lactate metabolism-related genes in BC and TC. **(E)** Differentially expressed genes related to lactate metabolism in BC. **(F)** Differentially expressed genes related to lactate metabolism in TC. **(G)** Lactate metabolism-related genes with significant mutations in BC and TC. **(H)** Copy number variations in lactate metabolism-related genes in BC and TC.

First, we performed ssGSEA of lactate metabolism scores based on gene expression data from TCGA-BRCA and TCGA-THCA datasets to compare differences between tumor and normal samples. Lactate metabolism scores were significantly different between the two cancer types of lactate metabolism scores (*p* < 0.001, Figure 1A), with BC showing a higher lactate metabolism score than TC (Figure 1A). Subsequently, we compared the lactate metabolism scores between the two cancer types and the normal samples. The results illustrated significantly different lactate metabolism scores between the normal samples and both cancers (Figures 1B,C), where BRCA showed a higher score, while THCA displayed a lower score compared to normal samples. In addition, the distribution of expression levels of 245 lactate in patients with the two cancer types is shown in Figure 1D. Among those 245 genes, BRCA and THCA showed different expression trends (Figures 1E,F). Thus, we analyzed the mutations of genes related to lactate metabolism in BRCA and THCA and found that, in addition to mutations of the tumor suppressor gene *TP53*, genes such as *LYST* and *RB1* were also mutated (Figure 1G). Furthermore, analysis of copy number variations (CNVs) revealed more CNVs in BRCA and less in THCA (Figure 1H). These results demonstrate that lactate metabolism is correlated with tumorigenesis and shows significant tumor heterogeneity.

### 3.2 Lactate-Related Genes and Lactate Metabolism Scores Were Significantly Associated With the Survival of Patients With BRCA and THCA

**FIGURE 2:**
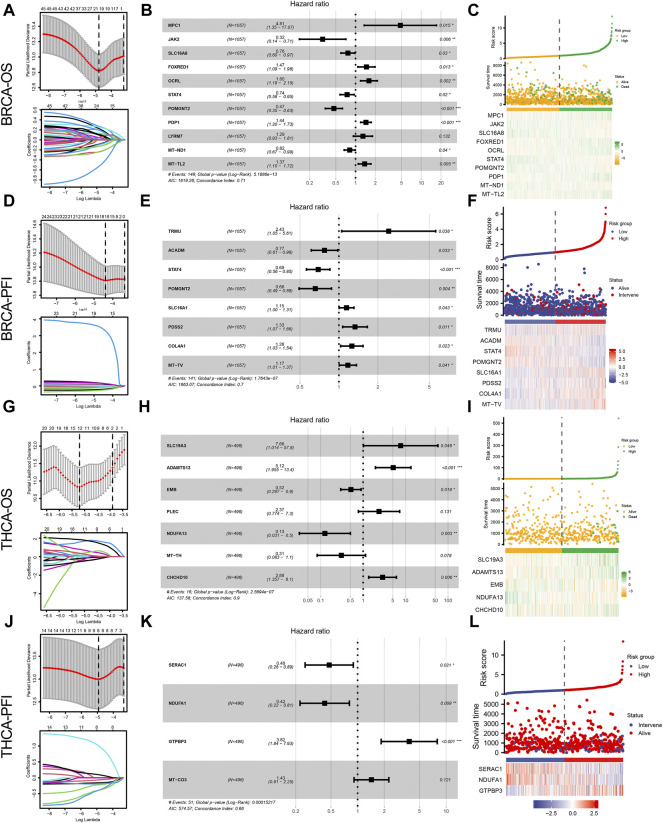
Lactate metabolism-related genes and prognostic analysis of patients with BRCA and THCA. **(A)** Lactate metabolism-related genes in BRCA and overall survival (OS)-related Lasso regression to screen for prognostic factors. **(B)** The forest map presents the results of a multivariate Cox regression between genes associated with lactate metabolism in BRCA and OS. **(C)** Heat map of OS-related independent risk factors in BRCA. **(D)** Lactate metabolism-related genes in BRCA and PFS-related Lasso regression to screen for prognostic factors. **(E)** The forest map shows the results of a multivariate Cox regression between lactate metabolism-associated genes in BRCA and PFS. **(F)** Heat map of PFS-related independent risk factors in BRCA. **(G)** Lactate metabolism-related genes in THCA and OS-related Lasso regression to screen for prognostic factors. **(H)** The forest map reveals the results of multivariate Cox regression between lactate metabolism-related genes in THCA and OS. **(I)** Heat map of OS-related independent risk factors in THCA. **(J)** Lactate metabolism-related genes in THCA and PFS-related Lasso regression to screen for prognostic factors. **(K)** The forest map indicates multi-factor Cox regression results of lactate metabolism-related genes and PFS in THCA. **(L)** Heat map of PFS-related independent risk factors in THCA.

To investigate the correlation between the expression of genes related to lactate metabolism and cancer survival, we first performed OS and PFS analyses. According to the median gene expression levels of the two cancer types, the patients were divided into high expression and low expression groups, respectively. We performed univariate Cox regression, Lasso regression, and multivariate Cox regression analyses to identify independent prognostic factors for genes associated with lactate metabolism in BC and TC. After analysis, we identified 10 OS-related independent prognostic factors in BRCA (Figures 2A–C) and 8 PFS-related independent prognostic factors (Figures 2D–F) and plotted a heat map of risk factors based on their expression. The genes *STAT4* and *POMGNT2* are independent prognostic factors in both OS and PFS. Furthermore, in THCA, we identified five OS-related independent prognostic factors (Figures 2G–I) and three PFS-related independent prognostic factors (Figures 2J–L) and plotted a heat map of risk factors based on their expression.

**FIGURE 3:**
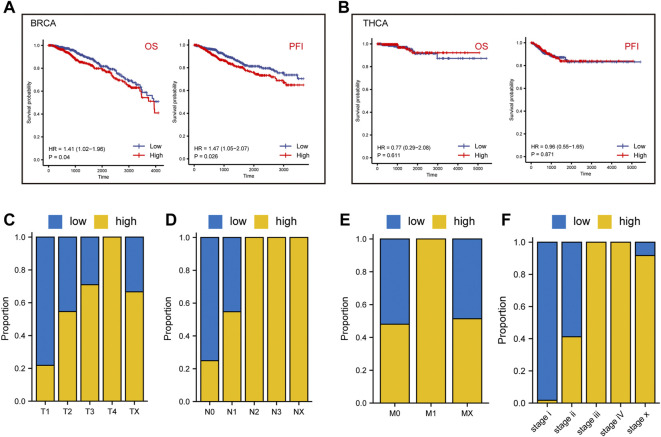
Lactate metabolism score and prognostic analysis of patients with BRCA and THCA. **(A)** The lactate metabolism score in BRCA signifies significant statistical differences in both overall survival (OS) and progression-free survival (PFS). **(B)** There are no statistically significant differences in the lactate metabolism score in THCA between OS and PFS. **(C–F)** The proportion of lactate metabolism scores in T, N, M, and the cancer stages in BRCA.

We further analyzed the prognostic value of BRCA and THCA in OS and PFS based on their lactate metabolism scores. As shown in Figure 3A, for BRCA, the lactate metabolism scores suggested significant statistical differences in both OS and PFS, and the prognosis of patients with high lactate metabolism scores was worse than that of patients with low lactate metabolism scores. In THCA, the lactate metabolism score did not differentiate OS from PFS, i.e., it could not be used as a prognostic biomarker of THCA (Figure 3B). We then compared the difference in cancer stages based on the lactate metabolism score of patients with BRCA. As shown in Figures 3C–G, high lactate metabolism scores were associated with higher T, N, M, and total cancer stages.

### 3.3 Validation of Gene Expression in Gene Expression Omnibus Datasets

**FIGURE 4:**
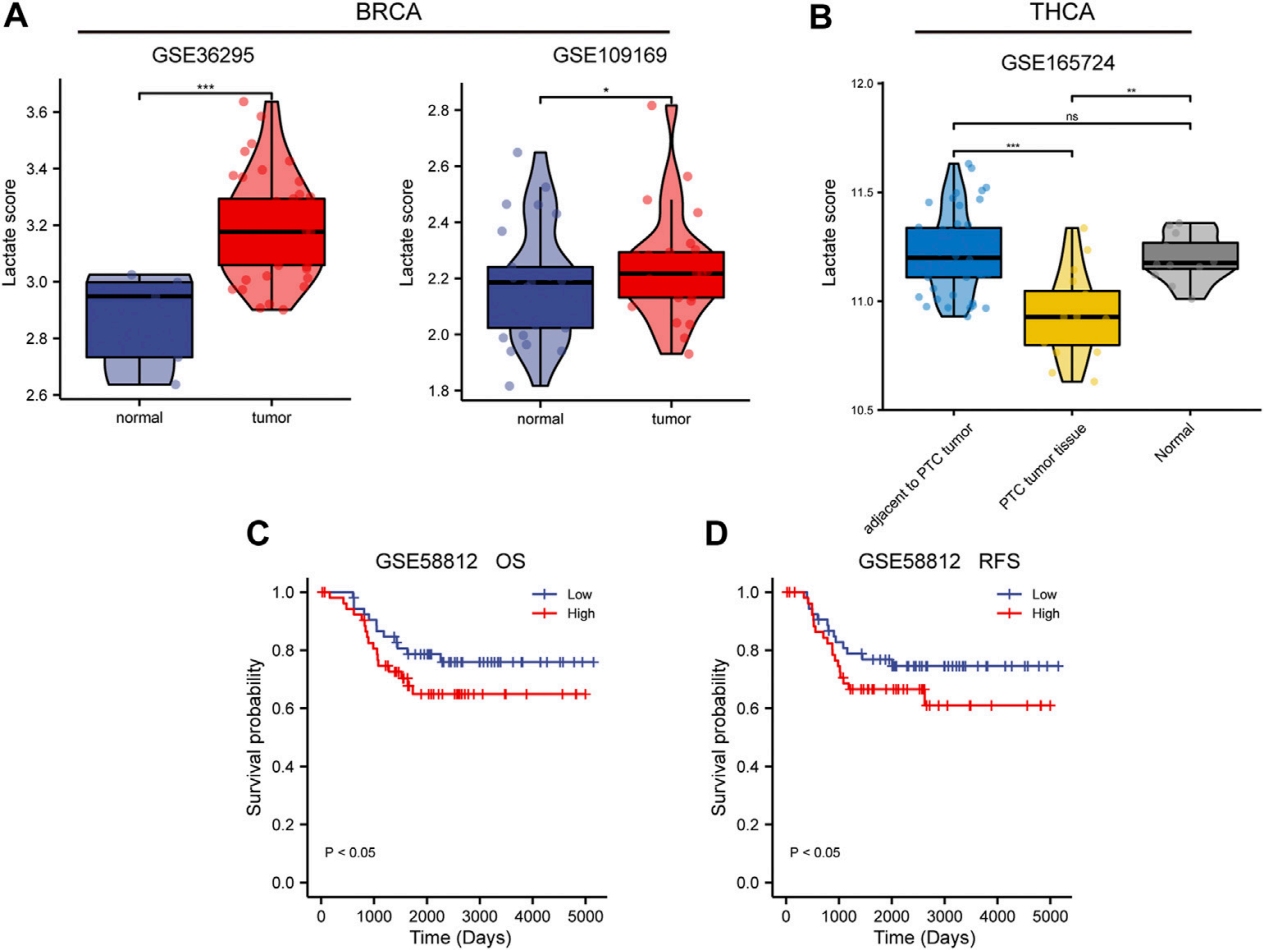
Validation of lactate metabolism scores by GEO database. **(A)** Comparison of lactate metabolism scores in normal and neoplastic tissues from the breast cancer (BC) datasets GSE36295 and GSE109169. **(B)** Comparison of lactate metabolism scores in normal, paracancerous, and thyroid cancer (TC) tissues from the TC dataset GSE165724. **(C)** The lactate metabolism score in the BC dataset GSE58812 was significantly correlated with the overall survival (OS). **(D)** The lactate metabolism score in the BC dataset GSE58812 was significantly correlated with the recurrence-free survival (RFS).

To validate the differential and prognostic results of the lactate metabolism scores described above, we selected appropriate BC and TC datasets from the GEO database for verification. First, we demonstrated the difference in lactate metabolism scores between normal and neoplastic tissues from BC and TC. In the BC datasets GSE36295 and GSE109169, the lactate metabolism score in tumor tissues were significantly higher than that TCGA-BRCA dataset (Figure 4A). In the TC data set GSE165724, the lactate metabolism score in the tumor tissue was significantly lower than that in normal and paracancerous tissues (Figure 4B), which was also consistent with the results from the previous TCGA-THCA dataset. Patients with high lactate metabolism scores had a poorer prognosis of OS and RFS in the GSE58812 BC dataset, according to a subsequent prognostic analysis—a finding that was consistent with prior TCGA-BRCA prognostic studies (Figures 4C,D).

### 3.4 Lactate Metabolism Scores Were Associated With Human Leukocyte Antigen, Tumor-Infiltrating Lymphocyte, and Infiltration, and Interferon in Breast Cancer and Thyroid Cancer

**FIGURE 5:**
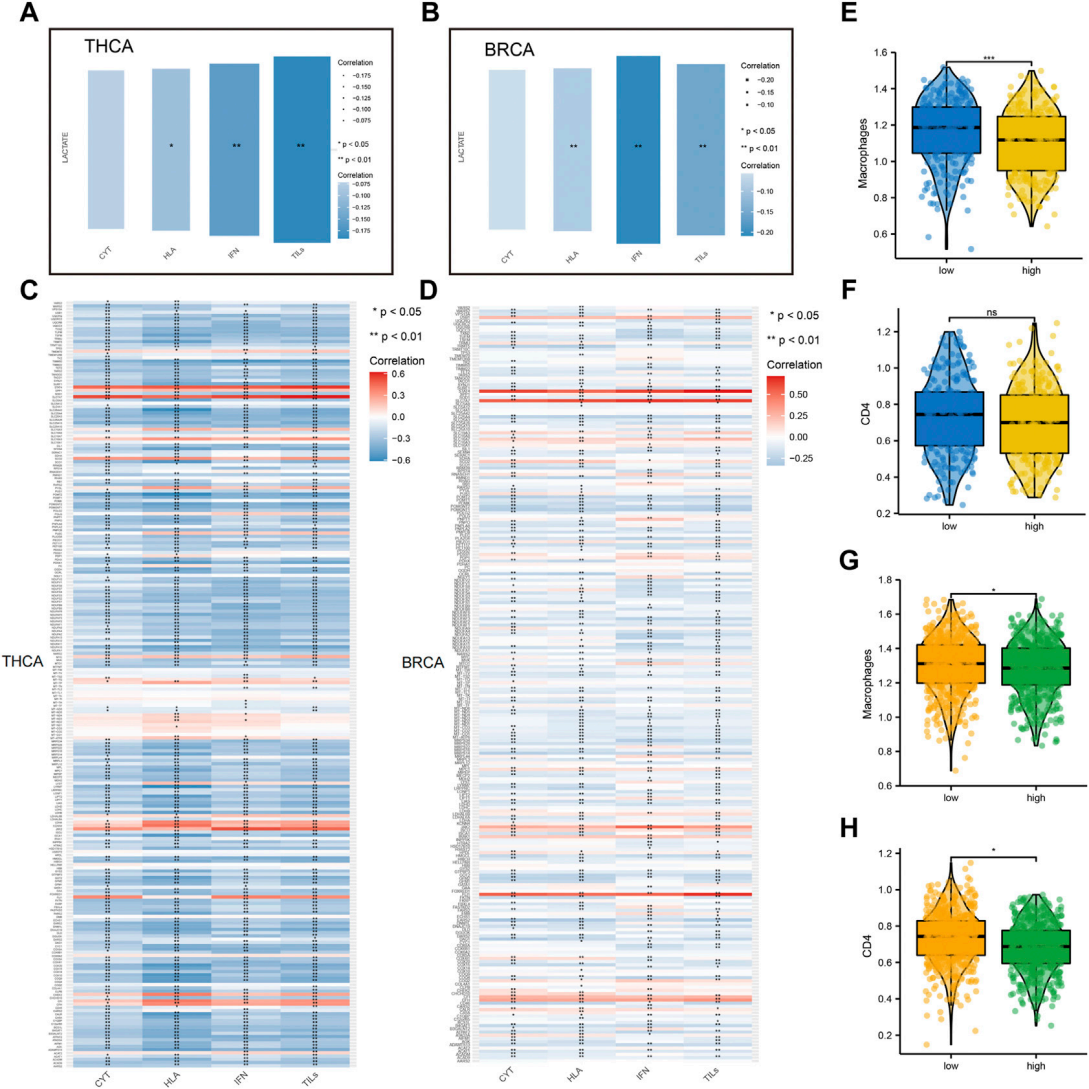
Relationship between lactate metabolism scores and tumor immunity in breast cancer and thyroid cancer. **(A,B)** Relationship between lactate metabolism scores in THCA and BRCA and expression of immune cytolytic activity (CYT), interferon (IFN), tumor-infiltrating lymphocytes (TILs), and human leukocyte antigen (HLA). **(C,D)** The relationship between the expression of lactate metabolism-related genes in THCA and BRCA and that of CYT, IFN, TILs, and HLA. **(E,F)** Differences in the infiltration of macrophages and CD4+T cells in THCA between high and low lactate metabolism score groups. **(G,H)** Differences in the infiltration of macrophage and CD4+T cells in BRCA between high and low lactate metabolism score groups.

To further explore the correlation between lactate metabolism and tumor immunity, we included multiple immune-related indicators, including CYT, HLA expression, IFN response, and TIL infiltration. Notably, we found a high correlation of lactate metabolism scores with HLA expression, TIL infiltration, and IFN response in BC and TC (Figures 5A,B). In addition to comparing the correlation between the lactate metabolism score and the above indicators, we studied the correlation between the lactate metabolism-related genes and the indicators. The results revealed that most of the lactate metabolism-related genes were negatively correlated with the indicators; however, a few genes, such as *STAT four* and *SLC7A7*, were positively correlated, with approximately the same trend in BRCA and THCA (Figures 5C,D).

Considering the apparent correlation between the lactate metabolism score and tumor immunity, we analyzed more differences in macrophage and CD4+T cell infiltration between the high and low lactate metabolism score groups for BRCA and THCA. In TC, macrophage infiltration was significantly higher in the low lactate group than in the high lactate group. However, CD4+T cell infiltration was not significantly different between the two groups (Figures 5E,F). In BC, macrophage and CD4 + T cell infiltration in the high lactate group were lower than those in the low lactate metabolism score group (Figures 5G,H). The above results suggest that lactate metabolism is negatively correlated with immune infiltration, i.e., under high lactate metabolism, immune cell infiltration is reduced.

### 3.5 The Lactate Metabolism Score Is a Good Predictor of Immune Characteristics in Thyroid Cancer and Breast Cancer

**FIGURE 6:**
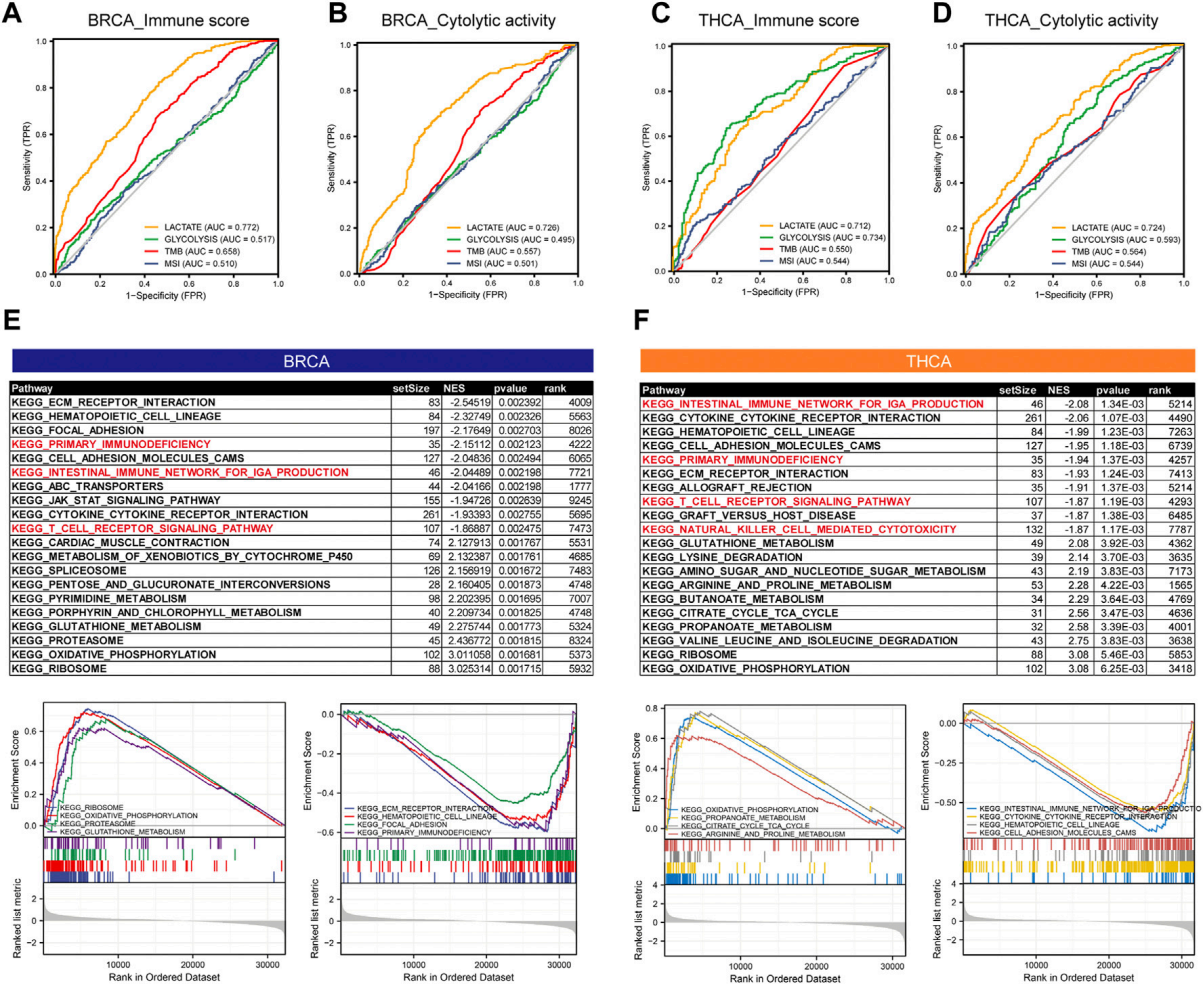
The lactate metabolism score is a good predictor of immune characteristics in thyroid and breast cancers. **(A,B)** Comparison of receiver operating characteristic (ROC) curves of lactate metabolism score, glycolytic activity in BRCA, and the ability of microsatellite instability (MSI) and tumor mutation burden (TMB) to predict the immune score and immune cytolytic activity (CYT). **(C,D)** Comparison of ROC curves of lactate metabolism score, glycolytic activity in THCA, and the ability of MSI and TMB to predict the immune score and immune CYT. **(E,F)** The KEGG enrichment pathways of lactate-related genes in BRCA and THCA.

Given the previous implications, we observed a significant correlation between lactate metabolism and tumor immunity. To determine whether the lactate metabolism score could predict tumor immunity, we used ROC curve analysis to assess the contribution of the lactate metabolism score in predicting two immune characteristics (immune score and immune CYT). Patients were divided into high and low groups according to the median immune characteristic score of each cancer type. Moreover, to compare the predictive power for tumor immunity, we included TMB, MSI, and glycolytic characteristics in this analysis. Compared with TMB and glycolysis scores, we observed a higher probability of success for lactate metabolism score as a predictor of the immune score and a lower probability of success for MSI as a predictor (Figures 6A,B). The lactate metabolism score in THCA also indicated the possibility of good prediction, and the glycolysis score provided a better prediction of the immunological features of THCA, but TMB and MSI have a low predictive performance (Figures 6C,D)lactate metabolism score.

To investigate the potential mechanism of lactate metabolism scores for estimating immune activity, we compared gene expression profiles between groups with high and low lactate metabolism scores and identified KEGG enrichment pathways in each of the two cancer types by GSEA. In both cancer types, the immune-related pathways, such as primary immunodeficiency, the intestinal immune network for IgA production, and the T-cell receptor signaling pathway, were downregulated in the high lactate group (Figures 6E,F).

In summary, the above results suggest some of these pathways play a key role in linking tumor lactate metabolism to tumor immunity.

### 3.6 Lactate Metabolic Score and Drug Sensitivity Analysis in BRCA and THCA

**FIGURE 7:**
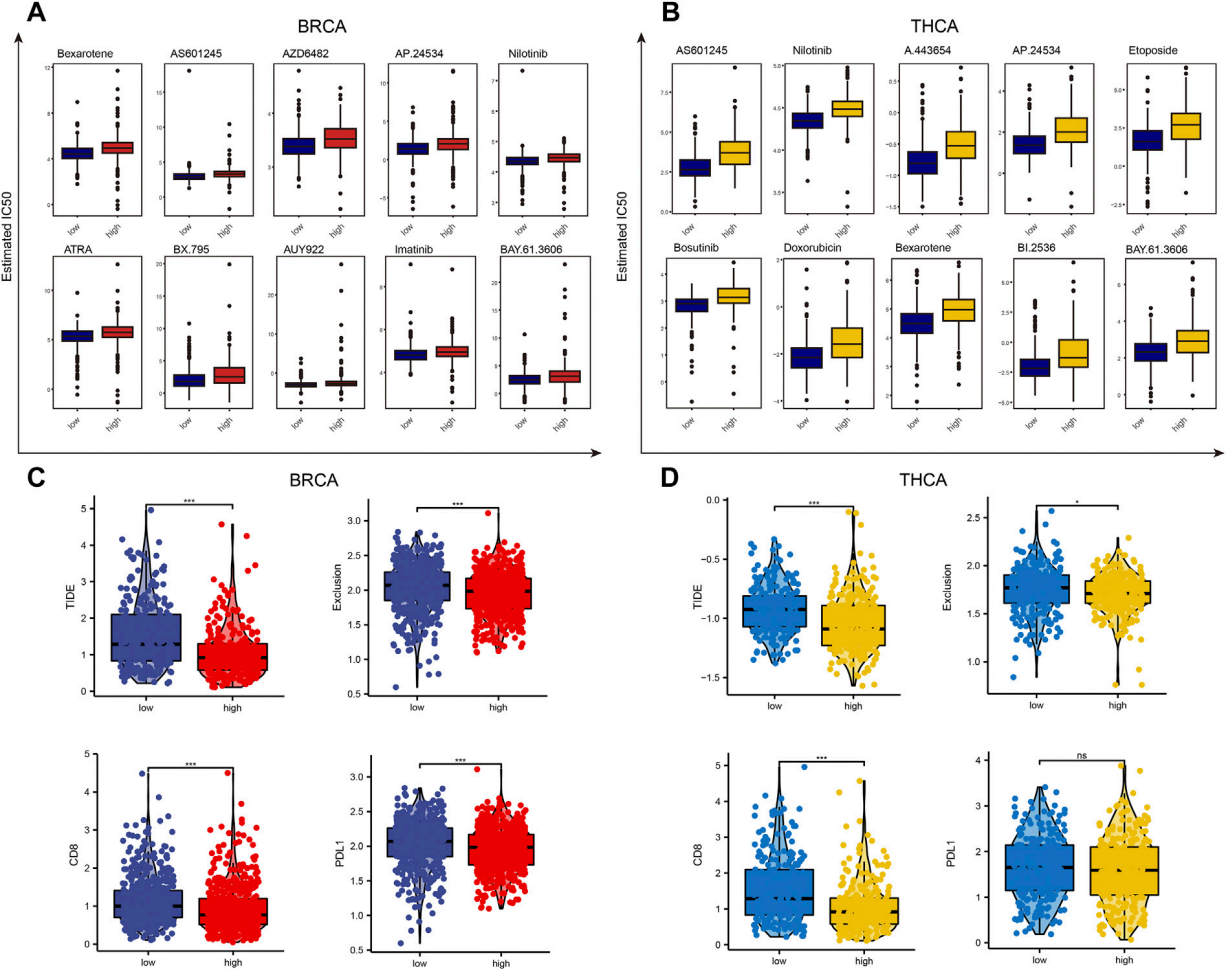
Drug sensitivity analysis of patients with high and low lactate metabolism scores of breast cancer and thyroid cancer. **(A,B)** The GDSC database predicts the difference in sensitivity of patients with high and low lactate metabolism scores to different anticancer drugs, and 10 drugs with the most significant difference are ranked according to the *p*-values. **(C,D)** The difference in the susceptibility of patients with high and low lactate metabolism scores to immunotherapy and associated biomarkers as predicted by the TIDE algorithm.

First, we assessed the difference in susceptibility to common antineoplastic agents between the high and low lactate metabolism score groups using the GDSC database. In BC, patients with a low lactate metabolism score were highly sensitive to most anti-tumor drugs, such as Bexarotene, AS601245, and AZD6482. Similarly, in TC, patients with a low lactate metabolism score were highly sensitive to anti-cancer drugs (Figures 7A,B).

Given the important role of immunotherapy in tumors at present, we first evaluated the sensitivity of patients in the high and low lactate metabolism score groups to immunotherapy using the TIDE algorithm. As shown in Figure 7C, the expression of the TIDE e, exclusive score, CD8, and CD274 scores in BC was lower in the high lactate group than in the low lactate group, i.e., patients in the high lactate group might be more sensitive to the immunotherapy. The same trend was seen in patients with TC. However, PD-L1 expression was not significantly different between the high and low lactate groups (Figure 7D). These results suggest that patients with high and low lactate metabolism scores from different tumors may have varied drug sensitivities to common anticancer drugs and immunotherapy, indicating the presence of tumor heterogeneity.

## 4 Discussion

Despite great advances in diagnosis and treatment, both BC and TC are solid tumors with high incidence and heterogeneity (Holm et al., 2017; Deng et al., 2020). The complexity of the TME in BC sand TC, including the accumulation of lactate, leads to inadequate therapeutic response and drug resistance, and some patients relapse during treatment. Lactate can act as both a metabolic fuel for oxidative cells and a signaling molecule in the TME, which is responsible for several invasive characteristics of cancer cells, including proliferation, invasion, angiogenesis, immune evasion, and therapy resistance. New markers are therefore urgently needed for the better management of both diseases. In this study, we constructed a lactate metabolism score based on lactate metabolism-related genes and established stable and precise features for prognosis prediction and comprehensive treatment of BC and TC patients.

Although there have been many previous studies investigating the association between lactate and tumors, this study is still the first study to quantify lactate metabolism based on ssGSEA in BC and TC simultaneously and perform a comprehensive analysis of molecular and clinical characteristics. We found significant differences in lactate metabolism scores between the two cancers in normal tissues, indicating that lactate metabolism scores may not be a prognostic marker in TC. We found that few studies have confirmed that the lactate level of TC tissues is significantly lower than that of normal tissues after reviewing the literature. One of the most important reasons to explain this phenomenon is that changes in only one metabolite are not sufficient to predict the development, metastasis, and immunosuppression of TC (Khatami et al., 2019). Zhao et al. demonstrated that glucose metabolism cannot be the only important metabolite because the metabolism of lipids, amino acids, and nucleic acids is important as well (Zhao et al., 2015). In addition, several metabolomics studies have confirmed that the levels of various cancer metabolites such as fatty acids, glutamine, lysine, lactate, taurine, and leucine together constitute biomarkers for TC (Wojtowicz et al., 2017; Zhou et al., 2017). It is also worth mentioning that we found a high correlation between lactate metabolism scores with HLA expression, TILs infiltration, and IFN response in BC and TC. In addition, our study addressed the use of lactate metabolism scores as an evaluation of anti-tumor drug sensitivity, which also indicated a positive correlation between targeting TME and the effectiveness of multiple immunotherapies. At the same time, we understand that the absence of further basic experiments is one of the limitations of this article. Since patients with low lactate scores often achieve better prognosis and immunotherapeutic response, further experiments are helpful to elucidate the molecular mechanism by which lactate inhibits TME immunity.

The predictive role of the lactate metabolism score in BC and TC is quite different. In our study, BRCA had higher lactate metabolism scores than normal tissues, while THCA had lower lactate metabolism scores than normal tissues, both of which showed the same trend in copy number variation (CNV) analysis. Meanwhile, the lactate metabolism score did not differentiate OS from PFI in THCA. The above analysis was also validated in the GEO database. Although immune cell infiltration is reduced in both cancers under conditions of high lactate metabolism, CD4 + T cell infiltration was not correlated with lactate metabolism score in TC. Lactate metabolism scores are both good predictors of immune characteristics and drug sensitivity in BC and TC.

Recent studies suggest the role of lactate metabolism-related genes in predicting tumor progression and response to immunotherapy. Xie et al. demonstrated that the enhanced expression of four lactate risk-related genes, *SLC25A3*, *HPDL*, *NDUFA13*, and *NARS2*, was correlated with poor prognosis in patients with skin cutaneous melanoma (SKCM), while patients with risk-related gene expression may benefit more from immune checkpoint inhibitor (ICI) therapy (Xie et al., 2022). Sun et al. established a lactate-related prognostic signature (LRPS) for the prognosis of patients with kidney renal clear cell carcinoma (KIRC) based on lactate metabolism-related genes and confirmed that LRPS might be effective in predicting the prognosis of patients with KIRC and that patients with low FPB1 and HDAH expression but high TYMP expression had a poor prognosis (Sun et al., 2022). In our study, we found that STAT4 was an independent prognostic factor for BC in terms of both OS and PFS. The signal transducer and activator of transcription 4 (STAT4), a member of the STAT family, increases Th1 cell differentiation and IFNγ production in immune cells and regulates tumor cell migration and proliferation (Yang et al., 2020). Although high STAT4 expression is a favorable prognostic factor in hepatocellular, breast, and ovarian cancers (Wang et al., 2015; Zhao et al., 2017; Wang et al., 2018), the exact role of STAT4 in cancer remains unclear. Tumor-infiltrating immune cells, an important component in the TME, are affected by lactate metabolism levels (Watson et al., 2021). Brand et al. proposed a mechanism of lactate-mediated immunosuppression, i.e., lactate and tumor acidosis inhibit nuclear factor of activated T cells (NFAT), a key activating transcription factor in tumor-infiltrating CD8 + T cells and NK cells, resulting in reduced IFNγ production (Brand et al., 2016). Ping et al. found a significant increase in intracellular lactate levels in tumor-infiltrating lymphocytes in the TME of gastric cancer. Increased lactate metabolism levels were inversely correlated with the percentage of TH1 cells and CTLs in the tumor, reflecting altered and impaired immune competence within the TME (Ping et al., 2018). Macrophages have two central polarization states, including M1 and M2 (Wynn et al., 2013). M1 macrophage polarization contributes to immunity against tumors, whereas lactate favors TAM polarization toward a pro-tumor M2 phenotype (Chen et al., 2021). Lactate in the TME interacts with a pH-sensing membrane receptor, Gpr132, on macrophages. Activation of this receptor can increase the expression of M2 polarization-related genes (Chen et al., 2017). Moreover, regulation of lactate levels can redistribute M2-TAM and upregulate PD-L1 to facilitate tumor immune escape, revealing macrophages as a “checkpoint” in organisms (Shan et al., 2020). Our findings followed a trend that showed lactate metabolism was inversely connected with immune infiltration and that patients with high lactate metabolism scores would benefit more from immunotherapy.

## 5 Conclusion

This is the first study to comprehensively evaluate the characteristics of lactate metabolism-related genes in BC and TC and construct a novel lactate metabolism score, which has a high value for predicting prognosis and reflecting immune responses in BC and TC. The lactate metabolism score is a predictive factor that is closely linked to clinical outcomes. In addition, patients with different scores have different TME statuses. Therefore, the lactate metabolism score is a promising prognostic signature for assessing the molecular and immune characteristics of BC and TC, which can provide important insights for subsequent functional study mechanisms and guide clinicians to make rational treatment decisions.

## Data Availability

The datasets presented in this study can be found in online repositories. The names of the repository/repositories and accession number(s) can be found in the article/Supplementary Material.
